# Acute Cervical Disk Herniation Resulting in Sudden and Severe Neurologic Deterioration: A Case Series

**DOI:** 10.1055/s-0036-1593357

**Published:** 2016-09-17

**Authors:** Ran Harel, Nachshon Knoller

**Affiliations:** 1 Spine Surgery Unit, Talpiot Medical Leadership Program, Department of Neurosurgery, Sheba Medical Center, affiliated to Sackler Medical School, Tel-Aviv University, Tel-Aviv, Israel

**Keywords:** acute cervical herniated disk, acute neurologic deterioration, Brown-Séquard syndrome, cervical myelopathy, anterior cervical approach

## Abstract

**Objective** Nontraumatic acute cervical disk herniation resulting in acute severe neurologic deficit is a rare entity described in a limited number of case reports. We describe the management and outcome in patients presenting with severe neurologic deterioration caused by acutely herniated cervical disks.

**Methods** Four patients (mean age 39.5 years) presented to our tertiary care academic medical center from September 2012 to September 2013 with severe progressive neurologic deficits due to cervical disk herniation and were included in the series. Patients' surgical, medical, and imaging records were retrospectively reviewed under an Institutional Review Board waiver of informed consent.

**Results** Patients in the series presented with acute neurologic deterioration, including paraparesis, Brown-Séquard syndrome, or quadriparesis deteriorating to quadriplegia. Emergent magnetic resonance imaging (MRI) scans and emergent decompression and fusion for acute soft disk herniation were performed in all cases. All patients recovered to excellent functional status with Frankel score improvement from B (one patient)/C (three patients) to E (three patients)/D (one patient).

**Conclusions** Acute cervical disk herniation with acute neurologic deterioration is a medical emergency necessitating emergent MRI and surgical decompression. Clinical presentation varies. In patients with rapid-onset neurologic deterioration, a high level of suspicion for this rare entity is indicated.

Cervical disk disease usually causes cervical myelopathy or radiculopathy with a gradual stepwise neurologic deterioration. 1 Acute cervical disk herniation resulting in rapid onset neurologic deficit, unrelated to trauma or a neoplastic process, is rare. Acute cervical disk herniation as a cause for quadriparesis was described in seven case reports, 2 3 4 5 6 and additional two cases described in the Japanese literature were cited by Suzuki et al. 7 In addition, three cases of paraplegia caused by nontraumatic disk herniation 7 8 9 10 and five cases of Brown-Séquard syndrome were described. 11 12 We hypothesized that rapid diagnosis and treatment could improve the neurologic outcome of these patients. Raising the awareness of this diagnosis and enhancing the importance of magnetic resonance imaging (MRI) availability in emergent settings was our main objective.

We report presenting symptoms, treatment, and outcomes of patients with acute cervical disk herniation causing acute neurologic deteriorations treated in our medical center.

## Methods and Materials

Four patients (mean age 39.5 years, range 32 to 47) presented to our tertiary care academic medical center from September 2012 to September 2013 with progressive paresis due to cervical disk herniation and were included in the series. The patients' surgical, medical, and imaging records were retrospectively reviewed under an Institutional Review Board waiver of informed consent.

## Results

### Patient 1

**Fig. 1:**
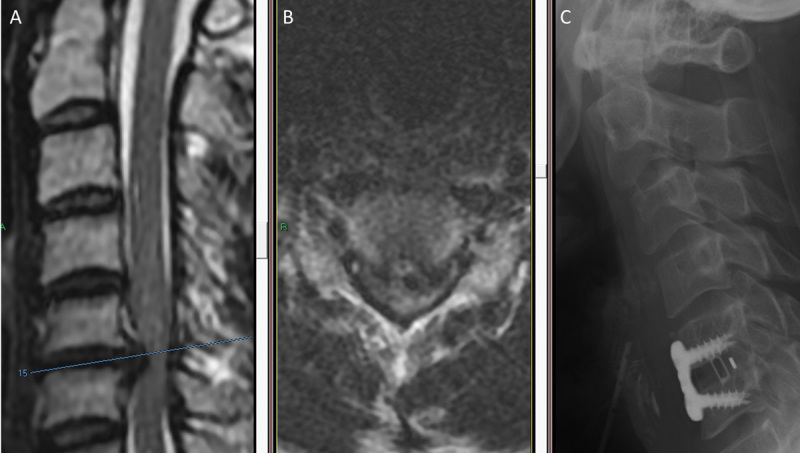
Acute disk herniation caused acute quadriparesis that rapidly progressed to quadriplegia during magnetic resonance imaging (MRI) examination in this 34-year-old man. (A) Sagittal and (B) axial T2-weighted MRI examinations demonstrate a central C5–C6 disk herniation with severe spinal cord compression. (C) Lateral X-ray demonstrates C5–C6 diskectomy and fusion with implantation of polyetheretherketone cage and fixed titanium plate.

A 34-year-old otherwise healthy man presented to the emergency room with quadriparesis. Three weeks prior to his admission, he had experienced neck pain and limb numbness. Physical examination revealed upper and lower extremity weakness (4/5), sensory level at T2, and hyperreflexia in all four limbs with clonus and positive Babinski sign in the lower extremities. Emergent cervical T2-weighted MRI revealed a huge C5–C6 herniated disk with spinal cord compression ( Fig. 1A, B ). Repeat neurologic examination after the MRI revealed weakness of the distal upper extremities (2/5) and lower extremities (0/5; Frankel grade B). Emergent C5–C6 diskectomy was performed, retrieving a huge soft herniated disk as the posterior longitudinal ligament (PLL) was opened. The diskectomy was followed with fusion with implantation of a polyetheretherketone (PEEK) cage and fixed titanium plate via anterior cervical approach ( Fig. 1C ). Following surgery, the patient recovered strength in his extremities (4 + /5 to 5/5). He was discharged to a rehabilitation facility 7 days later. At 3-month follow-up, he had a normal gait and full strength in his extremities with mild residual spasticity (Frankel grade E). An 18 months after surgery, examination revealed normal strength and sensation with slight spasticity, and bony fusion was noted on imaging.

### Patient 2

**Fig. 2:**
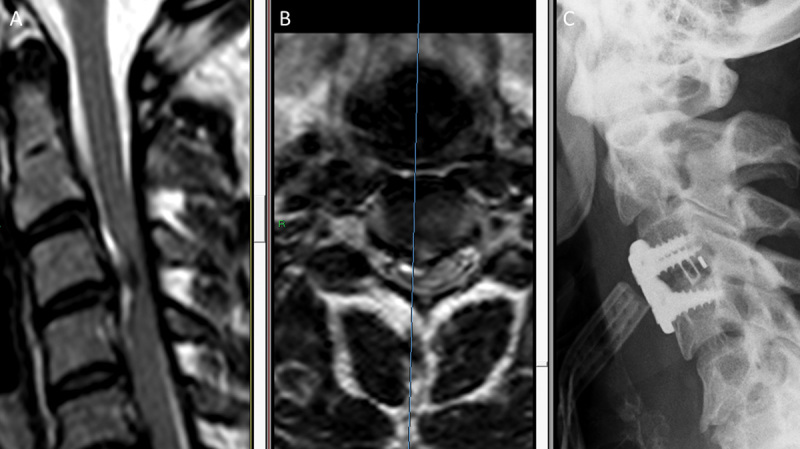
(A) Sagittal and (B) axial T2-weighted magnetic resonance imaging (MRI) scans demonstrate a right-sided acute disk herniation with spinal cord compression and cord deviation to the left in a 32-year-old man. (C) Lateral X-ray performed on postoperative day 1 following C3–C4 diskectomy and fusion with a polyetheretherketone cage and fixed titanium plate.

A 32-year-old man presented to the emergency room of another medical facility after awakening at 4 am with right hemiparesis. His medical history was unremarkable, although he had complained of neck pain 1 day before admission. Physical examination revealed severe hemiparesis of the right upper and lower extremities (3 +/5; Frankel grade C) and sensory disturbances on the left side (diagnosed as Brown-Séquard syndrome). Contrast-enhanced brain computed tomography (CT) and CT angiography were normal. Emergent cervical T2-weighted MRI revealed a C3–C4 right-sided disk herniation with marked spinal cord compression ( Fig. 2A, B ). The patient was transferred to our medical center for emergent C3–C4 diskectomy and fusion with a PEEK cage and fixed titanium plate ( Fig. 2C ). During surgery, a hole was recognized in the PLL, and a large soft sequestrated disk was removed from the epidural space. Following surgery, the patient recovered almost normal strength on the right side (5−/5) and was discharged home on postoperative day 2. At 2-month follow-up, he had achieved a full neurologic recovery with normal gate and extremity strength. At 8-month follow-up, he complained of slight neck pain, but his neurologic examination was normal (Frankel grade E). Bony fusion was evident on imaging.

### Patient 3

**Fig. 3:**
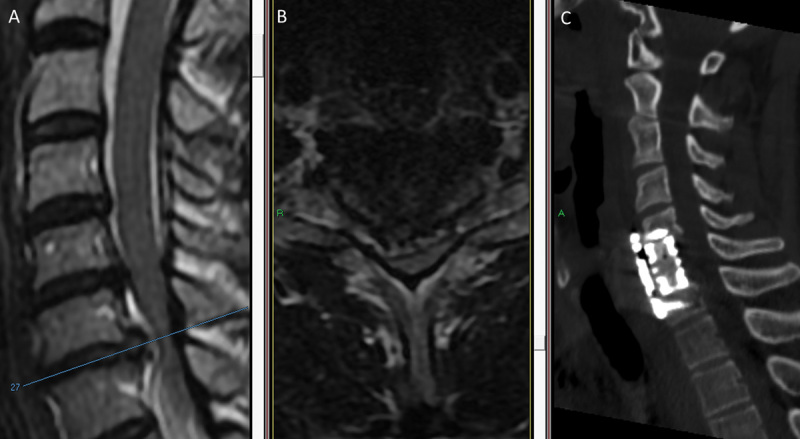
(A) Sagittal and (B) axial T2-weighted magnetic resonance imaging (MRI) examinations demonstrate acute C6–C7 disk herniation with a hyperintense signal in the spinal cord adjacent to the herniated disk. Cord compression is more severe on the right. (C) Postoperative computed tomography (CT) demonstrates implantation of a titanium mesh cage replacing the C6 body and dynamic plate.

A 45-year-old man presented to the emergency department unable to walk or stand. His medical history was notable for a history of whiplash 15 years earlier, and 3 days prior to his admission he had complained of lower extremity weakness after lifting a heavy object. Physical examination revealed bilateral leg weakness (3/5) and hyperreflexia in all four extremities with positive Babinski sign and a sensory level at T6 (Frankel grade C). Emergent T2-weighted MRI of the cervical and thoracic spine demonstrated a degenerated C5–C6 disk with herniation resulting in slight spinal cord compression; however, a huge C6–C7 herniation was seen with cranial disk migration and severe cord compression ( Fig. 3A, B ). During emergency surgery, C6 corpectomy was performed to extract the migrating C6–C7 disk and treat both degenerative levels. The PLL was opened and a soft herniated disk was retrieved. A mesh cage with local autogenous bone graft was inserted, and a dynamic plate was fixed with four screws ( Fig. 3C ). On postoperative day 1, the patient regained full strength in the lower extremities. On day 2, his gait was spastic and unsteady, but by day 4, his gait had improved to steady with only slight spasticity and he was discharged home. At 2-month follow-up, he had a normal gait, full strength in all extremities, and normal reflexes (Frankel grade E). At 13-month follow-up, he complained of neck pain and arm numbness, but physical exam demonstrated normal strength and sensation with lower limb hyperreflexia (Frankel grade E). Bony fusion was evident on imaging.

**Fig. 4:**
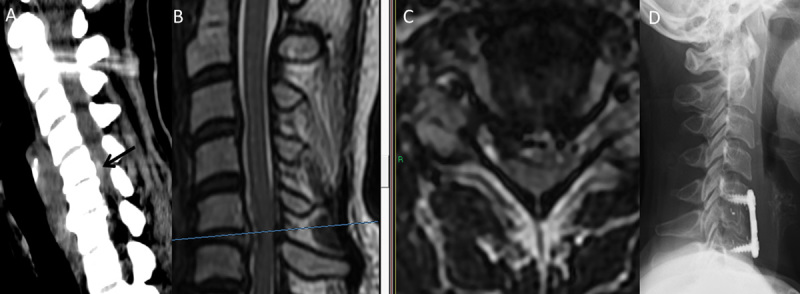
(A) Noncontrast admission computed tomography (CT) scan demonstrates C5–C6 disk herniation with canal stenosis (arrow) in a 47-year-old woman. (B) T2-weighted sagittal and (C) axial magnetic resonance imaging (MRI) scans performed 2 days later after neurologic deterioration reveal C5–C6 disk herniation with a hyperintense signal in the cord. (D) Postoperative lateral X-ray demonstrates a polyetheretherketone cage substitute for C6 vertebral body and anterior titanium plate.

### Patient 4

A 47-year-old woman presented to the emergency department complaining of cervical pain and inability to stand or walk. She had a history of Crohn disease, fibromyalgia, major depression, drug abuse, and borderline personality disorder. Independent examinations by a neurosurgeon, neurologist, and orthopedic surgeon produced concurring assessments of slight limb weakness, inconsistent with her complaints, and speculation that her difficulty in walking was of a nonorganic origin. A CT scan demonstrated C5–C6 disk herniation with spinal canal compromise ( Fig. 4A ). The patient was admitted for further workup, including cervical MRI with gadolinium. Neurologic examination the day after admission found her to be stable, and psychiatric evaluation suggested factitious simulation disorder; however, on the following day, neurologic evaluation was consistent with spastic quadriparesis and a reduction in extremity strength (4/5 proximal upper limbs, 3/5 distal limbs, 4/5 right lower limb, 2/5 left lower limb). The patient was hyperreflexic with positive Tromner, Hoffman, and Babinski signs, as well as sphincter incontinence (Frankel grade C). T2-weighted MRI of the spine revealed a C5–C6 herniated disk with marked compression of the spinal cord and a C6–C7 degenerative disk with mild compression and PLL ossification posterior to the C6 vertebra. A hyperintense signal within the cord was suggestive for possible sequelae of the compression ( Fig. 4B, C ). Urgent surgery via an anterior cervical approach was performed to achieve cord decompression with C6 corpectomy to resolve the adjacent segment and the PLL ossification. After PLL opening, two large fragments of soft disk were retrieved from the epidural space. The patient was fused with a PEEK cage with local autogenous bone graft and titanium dynamic plate ( Fig. 4D ). Following surgery, her neurologic examination improved. She was able to stand with assistance and regained partial sphincter control. She was transferred to a rehabilitation facility on day 16 with good recovery of strength in her extremities (4+/5). At discharge to her home a month after surgery, she could ambulate with the aid of a walker. At 2-month follow-up, she had recovered a normal gait, full sphincter control, and almost normal limb strength (5−/5) with slight hyperreflexia. At 6-month follow-up, her neurologic exam was unchanged (Frankel grade D). Imaging studies demonstrated the cage, plate, and screws in a steady position.

## Discussion

We present our experience in the management of four patients with no history of recent traumatic injury who presented with acute neurologic deterioration due to cervical disk herniation. Based on findings of cord compression on MRI, emergent surgery was performed, enabling all four patients to achieve a normal gait at 2- to 3-month follow-up. All patients were treated with spinal arthrodesis because the authors prefer motion restriction rather than motion preservation for patients with myelopathy. It is noteworthy that all patients in this series presented to the emergency room with only a partial deficit and were treated emergently.

Most acute cervical disk herniations are sequelae of traumatic injuries and are treated acutely as such. Degenerative spine disease is typically a chronic condition with gradual exacerbations. In 20 to 62% patients with cervical myelopathy, the condition deteriorates over 3 to 6 years 13 ; thus, degenerative disease is usually treated in an elective setting. Cervical disk herniation, unrelated to trauma, causing severe neurologic deterioration is rare. Suzuki et al described a case of cervical myelopathy presenting with a C6–C7 disk herniation and paraplegia in a 29-year-old man. 7 Although the patient had emergent MRI and surgery, he did not recover. Liu et al described a 75-year-old man presenting with nontraumatic disk herniation at C4–C5 and severe paraparesis. 9 The patient's strength improved after surgery, but he did not achieve a functional recovery.

Acute cervical disk presenting as acute Brown-Séquard syndrome is rarely described in case reports. 11 12 14 15 Abouhashem et al reported seven cases of disk herniation causing acute Brown-Séquard syndrome. 15 Their review described 45 cases of disk herniation resulting in hemisyndrome reported since 1928. Most of these patients had gradual deterioration, and only five patients had acute presentation diagnosed in the first week. Patient 2 is the sixth case of disk herniation with acute Brown-Séquard syndrome reported in the literature; however, in this case, symptom onset occurred during a night's sleep and the patient had a full recovery.

Acute quadriplegia resulting from cervical herniated disk was described by Sadanand et al in a 42-year-old patient with acute presentation after a sneeze. 4 This patient presented with a C4–C5 disk herniation resulting in complete C5 quadriplegia. Although he underwent emergent MRI and surgery, he remained quadriplegic and died during rehabilitation 18 days after surgery. Siam described a 48-year-old woman suffering from incomplete quadriparesis following a C5–C6 disk herniation. 2 The patient was operated within 6 hours following MRI scan and had good functional recovery.

In our series, patient 1 presented with quadriparesis that progressed to complete quadriplegia after admission MRI. The patient was operated within 3 hours of deterioration and recovered a normal gait and extremity strength. Perhaps because of her somewhat complex medical history, the walking difficulties of patient 4 were initially thought to be of a nonorganic origin despite examination by physicians from several subspecialties. Only after a rapid deterioration to Frankel grade C was the source of the problem correctly diagnosed. Fortunately, emergent surgery restored her neurologic function to near normal levels (Frankel grade D).

All patients in this series had symptoms of neck pain with mild motor or mild sensory disturbances prior to their severe neurologic deterioration. The time frame for these symptoms ranged from 1 day to 3 weeks prior to admission. Possible causes for these early symptoms could be injury to the annulus fibrosus or disk herniation with slight pressure on the spinal cord. We could not find any red flags to differentiate these patients from other patients in whom disk herniation led to a more typical presentation of gradual myelopathy progression.

All patients in this series underwent MRI examination within hours of the onset of marked neurologic deterioration, enabling urgent diagnosis and intervention. It is noteworthy that MRI may not be universally available on an emergency basis. The 2013 Organization for Economic Cooperation and Development report revealed that in 2011 the number of MRI scanners per capita varied widely, from 46.9/million population in Japan and 31.5/million in the United States to 10.8/million in Germany, 5.8/million in the United Kingdom, and only 2.5/million in Israel. 16 Hence, not all hospitals and spine centers are equipped with MRI scanners, and not all MRI scanners are operational 24-7.

Our study is limited by its retrospective nature and by the relatively small number of patients presented. These factors limit our ability to present statistical results to prove our hypothesis. However, to the authors' knowledge, it is the largest and only series of patients presenting with acute nontraumatic cervical disk herniation associated with spinal cord compression leading to sudden-onset rapid neurologic deterioration; thus, we believe the series emphasizes the importance of rapid diagnosis and intervention in this setting.

## Conclusion

Acute neurologic deterioration as a result of acute cervical disk herniation is a rare entity with variable presentation. The outcome in patients operated with incomplete deficits is better than surgery only after deterioration to a complete neurologic deficit; hence, rapid diagnosis and treatment are crucial. The capability to perform urgent MRI and surgery for patients presenting with neurologic deterioration should be mandatory for tertiary spine centers.
